# A Founder Pathogenic Variant of *PPIB* Unique to Chinese Population Causes Osteogenesis Imperfecta IX

**DOI:** 10.3389/fgene.2021.717294

**Published:** 2021-09-29

**Authors:** Wenting Zhu, Kai Yan, Xijing Chen, Wei Zhao, Yiqing Wu, Huanna Tang, Ming Chen, Jian Wu, Pengpeng Wang, Runju Zhang, Yiping Shen, Dan Zhang

**Affiliations:** ^1^ Women’s Reproductive Health Research Key Laboratory of Zhejiang Province and Department of Reproductive Endocrinology, Women’s Hospital, Zhejiang University School of Medicine, Hangzhou, China; ^2^ Department of Reproductive Endocrinology, Women’s Hospital, Zhejiang University School of Medicine, Hangzhou, China; ^3^ Department of Genetics and Reproduction, Women’s Hospital, Zhejiang University School of Medicine, Hangzhou, China; ^4^ Department of Genomic Medicine and Center for Medical Genetics, Changhua Christian Hospital, Changhua, Taiwan; ^5^ MyGenostics Inc., Beijing, China; ^6^ Division of Genetics and Genomics, Department of Neurology, Boston Children’s Hospital and Harvard Medical School, Boston, MA, United States; ^7^ Key Laboratory of Reproductive Genetics, Zhejiang University, Ministry of Education, Hangzhou, China

**Keywords:** osteogenesis imperfecta type IX, PPIB gene, founder mutation, pathogenic variant, Chinese

## Abstract

**Background:** Osteogenesis imperfecta (OI) is a heterogeneous genetic disorder characterized by bone fragility. *PPIB* pathogenic variants cause a perinatal lethal form of OI type IX. A limited number of pathogenic variants have been reported so far worldwide.

**Methods:** We identified a rare pedigree whose phenotype was highly consistent with OI-IX. Exome sequencing was performed to uncover the causal variants. The variant pathogenicity was classified following the ACMG/AMP guidelines. The founder effect and the age of the variant were assessed.

**Results:** We identified a homozygous missense variant c.509G > A/p.G170D in *PPIB* in an affected fetus. This variant is a Chinese-specific allele and can now be classified as pathogenic. We estimated the allele frequency (AF) of this variant to be 0.0000427 in a Chinese cohort involving 128,781 individuals. All patients and carriers shared a common haplotype, indicative of a founder effect. The estimated age of variant was 65,160 years. We further identified pathogenic variants of *PPIB* in gnomAD and ClinVar databases, the conserved estimation of OI type IX incidence to be 1/1,000,000 in Chinese population.

**Conclusion:** We reported a founder pathogenic variant in *PPIB* specific to the Chinese population. We further provided our initial estimation of OI-IX disease incidence in China.

## Introduction

Osteogenesis imperfecta (OI) is a group of hereditary connective tissue disorders that are caused by gene mutations that affect the quantity and/or quality of collagen which constitute the main framework of bones (Brusin, 2008; Hackley and Merritt, 2008; Forlino and Marini, 2016). Thus, OI has major manifestations in bone, leading to skeletal fragility, bowing deformities of long bones, and substantial growth deficiency (Marini et al., 2007). More than 20 different genetic types of OI had been reported, resulting in five clinical types (Zhytnik et al., 2020). The overall incidence of OI was estimated as from 1:5,000 to 1:20,000 per live birth without gender preference (Rauch and Glorieux, 2004; Forlino et al., 2011). The clinical presentation of OI-IX ranged from perinatal lethal to moderate osteogenesis imperfecta phenotypes (Pyott et al., 2011). Homozygous or compound heterozygous mutations in the *PPIB* gene had been indicated to cause OI-IX (8–9). To date, only a few pathogenic variants in *PPIB* have been reported. Initially, homozygous c.556–559delAAGA (p.Lys186GlnfsX8) and c.451C > T (p.Gln151X) variants were uncovered in two families (Van Dijk et al., 2009). Barnes et al. subsequently identified a homozygous c.2T > G (Barnes et al., 2010) and a homozygous c.563_566delACAG (p.Asp188Alafs*6) in OI-IX patients and their siblings. Jiang et al. reported the first Chinese OI-IX case with a compound heterozygous variant in *PPIB* (Jiang et al., 2017). Chang et al. reported two fetuses with a homozygous *PPIB* variant (c.509G > A/p.G170D) from the Taiwan region) (Chang et al., 2020).

Type IX is a severe form of OI; the *PPIB* gene encodes a 21-kDa protein cyclophilin B (CyPB), which catalyzes the rate-limiting step in collagen folding (Steinmann et al., 1991). CyPB also plays a role as a component of the collagen 3-hydroxylation complex and is involved in modification of types I, II, V, and XI collagen (Weis et al., 2010). CyPB deficiency would result in decreased 3-prolyl hydroxylation, post-translational overmodification, and slow collagen folding, leading to an autosomal-recessive OI (Krane, 2008).

In this study, we reported a rare but recurrent pathogenic variant in *PPIB* in the Chinese population. We further estimated the age of the founder mutation and the incidence of OI type IX in the Chinese population.

## Subjects and Methods

### Subjects

**FIGURE 1:**
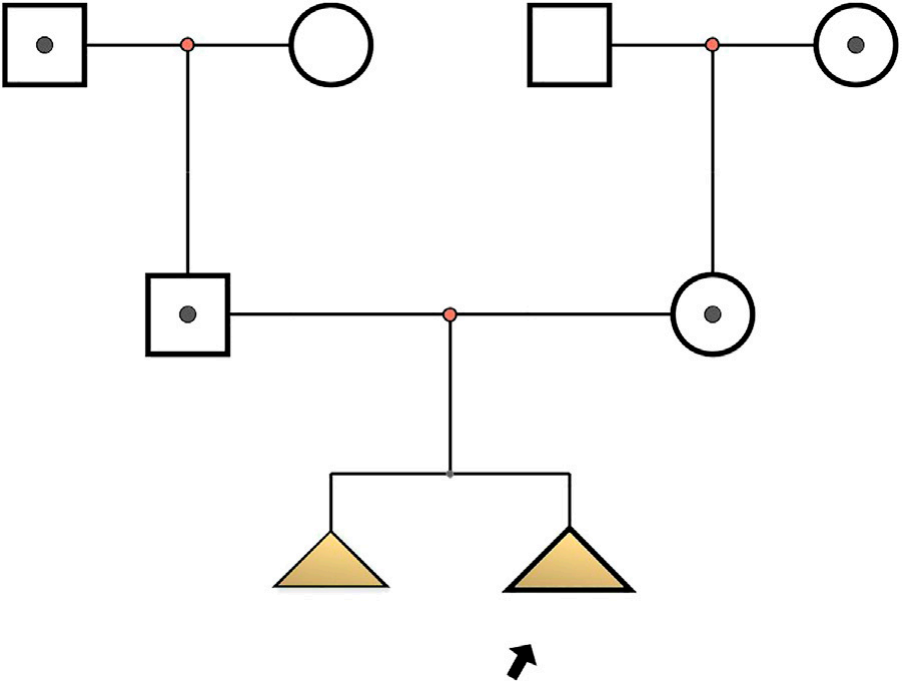
Pedigree of osteogenesis imperfecta IX-affected family. Arrow indicates the proband (Ⅲ2): c.509G > A in PPIB homozygote; I1, I4, II1, II2: c.509G > A heterozygote; Ⅲ1: with similar phenotype but no genetic examination.

**FIGURE 2:**
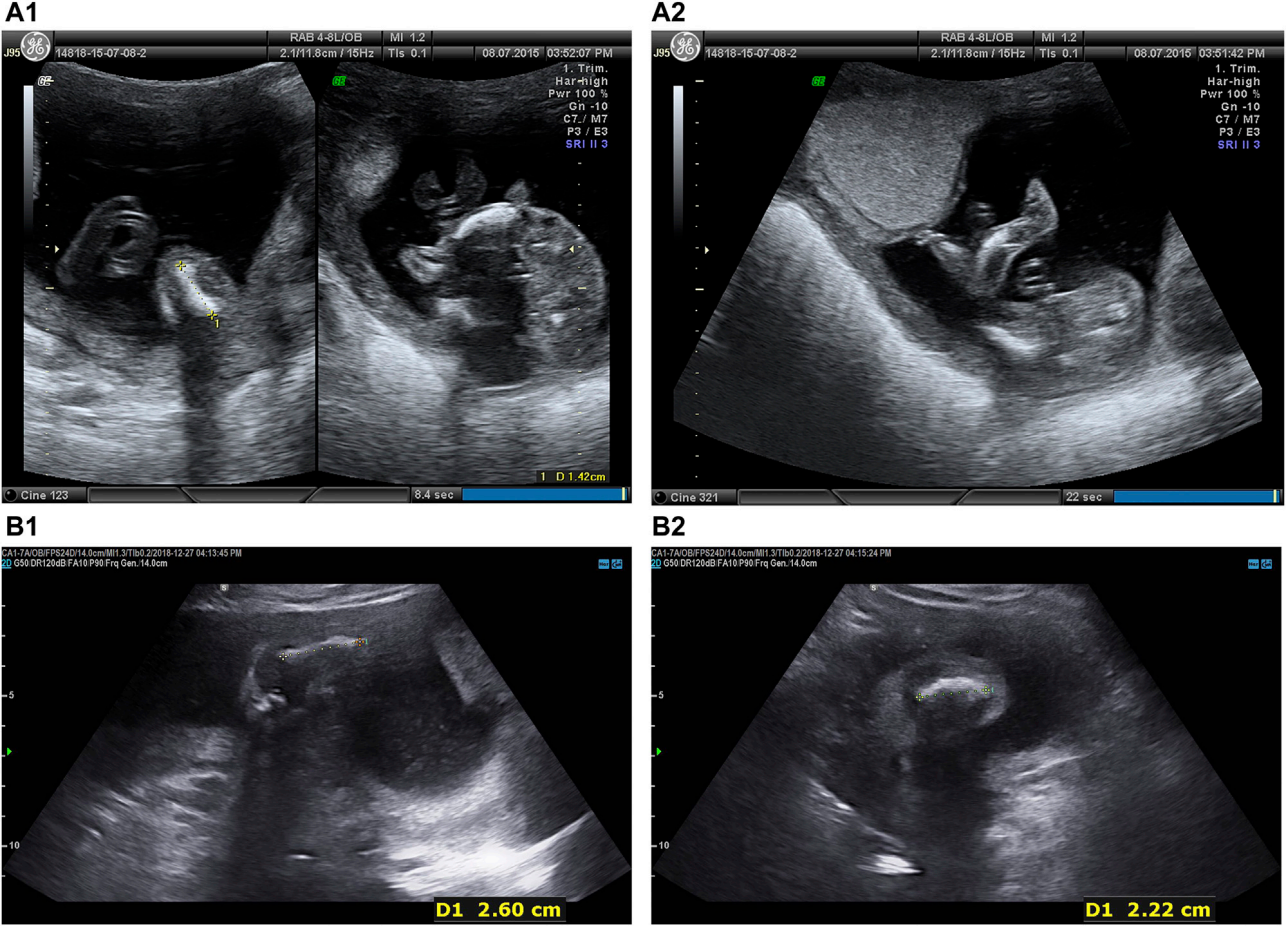
Prenatal ultrasonic examinations of the two fetuses in the second trimester showed bowed extremities **(A)** Intrauterine fetal ultrasonic examination of the first pregnancy at 18 + 5 weeks in 2015: navel, oval skull ring, double top diameter of 4.0 cm, lateral ventricle cerebellar hemisphere form with no obvious abnormalities, bowed lower extremities, bowed femur (1.4 cm), bowed tibia and fibula (1.4 cm), bilateral humerus (1.8 cm), radius (1.6 cm), bowed ulna (1.7 cm), crossroads of fetal heart breaks (0.2 cm), (A1 left) Femur. **(A1)** Tibia. **(A2)** Fibula.**(B)** Intrauterine fetal ultrasonic examination of the third pregnancy at 24 + 2 weeks in 2018: navel, oval skull ring, double top diameter of 5.6 cm, midline head circumference (20.4 cm) in the middle and lateral ventricle cerebellar hemisphere form with no obvious abnormalities, bowed upper and lower extremities, fetal bilateral bowed femur (1.8 cm), bowed tibia and fibula (1.9 cm), bowed humerus (2.6 cm), bowed radius (2.4 cm), bowed ulna (2.3 cm), fetal heart form with no obvious abnormalities, **(B1)** Femur. **(B2)** Fibula.

Two fetuses of similar prenatal ultrasonic phenotypes including bowed long bone of both the upper and lower limbs and short limbs from a Chinese couple were identified. Fetal femur and humerus lengths of the first fetus at 18+-week gestation were equivalent to those of a fetus at 14 + weeks, while the head circumference was equivalent to 17 + weeks. The fetal femur and humerus lengths of the second fetus at 24+-week gestation were equivalent to those of a fetus at 18 + weeks, while the head circumference was equivalent to 23 + weeks. Both parents and grandparents were normal and with no other family history of genetic bone disease. The pedigree and the results of prenatal ultrasonic examinations are shown in Figures 1, 2, respectively. This study was approved by the ethics committee of The Women’s Hospital affiliated to Zhejiang University (IRB-20210025-R).

### Whole-Exome Sequencing

WES was performed on the second fetus. DNA was extracted from the umbilical cord blood. The sample from the first fetus was not available. The average cover was 242.36X. Sequence data in the form of BAM files were generated *via* the Picard data-processing pipeline and contained well-calibrated reads aligned to the GRCh37/hg19 human genome reference. Samples across projects were then jointly called *via* the Genome Analysis Toolkit (GATK, http://www.broadinstitute.org/gatk) best-practice pipeline. The pathogenicity of the variants was interpreted according to the American College of Medical Genetics and Genomics/Association for Molecular Pathology (ACMG/AMP) guidelines (Richards et al., 2015). The variant detected by WES was validated by Sanger sequencing in the whole pedigree.

### Allele Frequency of the Variant

We examined the exome data of 128,781 Chinese anonymous individuals who took whole-exome sequencing test for various genetic conditions. We excluded the individuals biallelically affected for calculating AF.

### Haplotype Analysis and Statistical Analysis

Shared haplotype was delineated by identifying the homozygous SNPs surrounding the pathogenic variant detected in the proband. We included data reported in patients from the Taiwan region (Chang et al., 2020) as well as the SNP data of the 11 carriers found in the Chinese cohort. The founder effect was identified by taking individuals without the c.509G > A variant from the Chinese WES cohort as control. The Fisher’s exact test was used for statistical analysis (*p* < 0.001 was considered significant).

### Mutation Dating

We estimated the mutation age with a mutation dating web tool (https://shiny.wehi.edu.au/rafehi.h/mutation-dating), which uses the lengths of shared regions using an algorithm that makes use of recombination rates to predict the age of the most recent common ancestor (Gandolfo et al., 2014).

### Incidence of OI Type IX in Chinese Population

We further identified likely pathogenic or pathogenic variants of *PPIB* in gnomAD and ClinVar databases. The incidence of OI type IX was estimated in the Chinese population.

## Results

### Detection of a Pathogenic Variant

A homozygous variant (c.509G > A/p.G170D) was identified in *PPIB* by WES in the proband, and the variant was in heterozygous state in both parents and grandparents. The variant was validated by Sanger sequencing (Supplementary Figure S1). It is at the genomic location of chr15:64448943 and the exon 4 of *PPIB*. This variant had been detected in two fetuses in homozygous status (14) and in one fetus in compound heterozygous status (19), all in Chinese families.

The following evidence applies to the pathogenicity assessment according to the ACMG/AMP guideline (Table 1); thus, there is sufficient evidence supporting this variant as “Pathogenic.”

**TABLE 1:** Pathogenicity assessment.

Verdict	Applied rules	Evidence
Pathogenic	PM3	Three homozygous individuals reported (= a score of 1.5) (Chang et al., 2020)
	PM1	Change at the pro-isomerase domain
	PM2_P	Present only in the Eastern Asian population at a frequency of 17/18,394 (0.000924)
	PS3_P	Gene expression data (Jiang et al., 2017)
	PP3	REVEL score 0.94
	PP4	Consistent with OI-IX

### Estimation of the Allele Frequency, Founder Effect Analysis, and Mutation Dating

We identified 11 heterozygous carriers in the cohort of 128,781 Chinese (none suffered from any OI disorder). Thus, the overall AF of this variant in the Chinese population is 0.0000427.

We took advantage of our case with regions of homozygosity both at the upstream and downstream of the genomic location of the *PPIB* pathogenic variant c.509G > A and delineated the mutation-associated haplotype (Table 2). The two families reported from the Taiwan region and the 11 heterozygous carriers all shared the same SNPs surrounding the c.509G > A variant with our proband. The shared haplotypes were highlighted with blue color (Table 2). It appears there is a common breakpoint at Chr15: 64448365 in the two families from the Taiwan region, while breakpoints on the other side differed from case to case. As the coverage of WES was different, some positions of the haplotype of our proband were not tested in other patients and carriers. but the minimum common haplotype is certain, Chr15: 64448365–64792532.

**TABLE 2:** The shared haplotypes at the PPIB mutation site (position 6448943).

Chr15 position	REF	ALT	P1	P2	P3	P4	P5	P6	M1	M2	M3	M4	M5	M6	M7	M8	M9	M10	M11
59548475	C	T	T	T	T	T	T	T	T	T	T	T	T	T	T	T	T	T	T
59548821	G	A	A	A	—	A	A	—	—	—	—	—	—	—	—	—	—	—	—
59557518	C	A	—	—	—	—	—	A	—	—	—	—	—	—	—	—	—	—	—
59567634	T	C	C	C	—	C	—	T	—	—	—	—	—	—	—	—	—	—	—
59572821	C	CA	C	C	—	—	—	C	—	—	—	—	—	—	—	—	—	—	—
59606956	A	G	A	—	—	—	—	A	—	—	—	—	—	—	—	—	—	—	—
59607007	C	T	T	—	—	—	—	C	—	—	—	—	—	—	—	—	—	—	—
59654217	A	G	G	—	—	G	—	—	—	—	—	—	—	—	—	—	—	—	—
59655532	A	T	T	T	—	—	AT	—	—	—	—	—	—	—	—	—	—	—	—
59655578	C	T	T	T	—	—	—	—	—	—	—	—	—	—	—	—	—	—	—
59663443	G	A	A	A	—	G	—	G	—	—	—	—	—	—	—	—	—	—	—
59664479	A	G	G	G	G	G	G	G	—		—	—	—	—	—	—	—	—	—
59664607	C	A	A	A	A	A	A	A	A	—	A	A	A	A	A	A	—	A	—
59664963	G	C	C	C	C	C	C	C	C	—	C	—	—	—	—	—	—	—	—
63340647	A	G	G	G	G	G	G	G	G	—	G	G	G	—	G	G	G	G	G
63349096	G	A	A	A	A	A	A	A	A	A	A	—	A	A	A	A	A	A	A
63349564	C	T	T	T	T	T	T	T	T	—	T	—	—	—	—	—	—	—	—
63351488	G	A	A	A	A	A	A	A	—	—	—	—	—	—	—	—	—	—	—
63351687	A	G	G	G	G	G	G	G	G	—	G	G	G	—	G	G	G	G	G
63351840	C	A	A	A	A	A	A	A	A	A	A	A	A	A	A	A	A	A	A
63352092	G	A	A	A	G	A	—	A	—	—	—	—	—	—	—	—	—	—	—
63353760	C	A	A	A	A	A	A	A	A	—	A	A	A	A	A	A	A	A	A
63355275	A	T	T	T	T	—	T	T	—	—	—	—	—	—	—	—	—	—	T
63357852	T	G	G	G	G	G	G	G	—	—	—	—	—	—	—	—	—	—	—
63360564	G	A	A	A	—	—	—	—	—	—	—	—	—	—	—	—	—	—	—
63363401	C	CATTTTGTTTT	C	C	C	C	C	C	CATTTTGTTTT	CATTTTGTTTT	CATTTTGTTTT	CATTTT	CATTTTGTTTT	CATTTTGTTTT	CATTTTGTTTT	CATTTTGTTTT	CATTTTGTTTT	CATTTTGTTTT	CATTTT
63363654	A	G	G	G	—	—	G	G	—	—	—	—	G	G	G	G	G	G	—
63618110	A	C	C	C	C	—	C	C	—	—	—	—	C	C	C	C	C	C	—
63631327	G	T	G	G	G	G	G	G	—	—	—	—	—	—	—	—	—	—	—
63634405	G	T	T	T	T	T	T	T	—	T	—	—	—	—	—	—	—	—	—
63639016	A	G	G	G	G	G	G	G	G	G	G	—	G	G	G	G	G	G	—
63659262	C	C	C	C	—	—	—	—	—	—	—	—	—	—	—	—	—	—	—
63660537	G	A	A	A	—	A	A	A	—	—	—	—	—	—	—	—	—	—	—
63666420	C	A	A	A	—	A	A	—	—	—	—	—	—	—	—	—	—	—	—
64448019	T	TA	T	T	T	T	T	T	—	—	—	—	TA	TA	TA	TA	TA	TA	—
64448365	C	T	T	T	T	T	T	T	T	T	T	T	T	T	T	T	T	T	T
64448943	C	T	T	T	T	T	T	T	T	T	T	T	T	T	T	T	T	T	T
64792532	G	T	T	T	T	T	T	T	T	T	T	T	T	T	T	T	T	T	T
64808137	CAA	C	CAA	C	—	—	C	—	—	—	—	—	—	—	—	—	—	—	—
64835645	CT	C	C	C	—	—	—	—	—	—	—	—	—	—	—	—	—	—	—
64910945	G	T	—	T	—	T	—	T	—	—	—	—	—	—	—	—	—	—	—
64966403	G	T	G	G	G	G	G	G	—	—	—	—	—	—	—	—	—	—	—
65261931	T	C	T	T	T	—	T	T	—	—	—	—	—	—	—	—	—	—	—
65270512	G	A	A	A	—	—	—	A	—	—	—	—	—	—	—	—	—	—	—
65273075	CAACA	C	C	C	C	C	C	C	—	C	—	—	C	C	C	C	C	C	—
65273399	T	C	C	C	C	C	C	C	C	C	C	C	C	C	C	C	C	C	C
65273595	G	A	A	A	—	—	—	G	—	—	—	—	—	—	—	—	—	—	—
65298672	G	A	A	A	A	A	A	A	A	—	A	A	—	—	—	—	—	—	—
65315961	T	C	C	C	C	C	C	C	C	C	C	C	C	—	C	C	C	C	C

REF indicates GRCh37/hg19 human genome reference. ALT indicates the cDNA alteration. P1–P3 indicate patient, mother, and father of family 1 from Taiwan, respectively. P4–P6 indicate patient, mother, and father of family 2 from Taiwan, respectively. M1–M11 indicate the 11 carriers in the Chinese cohort of 128,781 individuals. “—” indicates not detected. The shared haplotypes are highlighted with blue color.

This variant is not present in other populations in the gnomAD database, but are present both in the Taiwan region and the Mainland of China, thus, a Chinese specific mutation. All patients and carriers shared a common haplotype. The deCODE genetic map shows there is no recombination hot spot in the region of common haplotype. A significant linkage disequilibrium (*p* < 0.001, Fisher’s exact probability test) was observed in the common haplotype. Together, these data suggested that the c.509G > A variant originated from a single founder.

We utilized the shared haplotypes for estimating the oldest age of founder mutation using the mutation dating software (https://shiny.wehi.edu.au/rafehi.h/mutation-dating/) (Gandolfo et al., 2014). The result showed that the founder mutation is estimated to be 3,258 generations old (95% confidence interval: 834–16,086 generations), or 65,160 years old (95% confidence interval: 16,680–321,720 years) assuming 20 years per generation.

### Estimation of the Incidence of Osteogenesis Imperfecta IX

We ascertained reported PPIB P/LP variants from databases (gnomAD and ClinVar) and recorded their AF in the Eastern Asian (EA) population (Table 3) the additive AF is 0.0010874; thus, the estimated disease incidence is about 1/1,000,000.

**TABLE 3:** Reported P/LP variants in database and their allele frequency in Eastern Asia.

No	Name	AF in EA	Pathogenicity
Missence			
1	c.509G > A	0.000924	Pathogenic
2	c.313G > A (p.Gly105Arg)	0.0000544	Pathogenic
3	c.26T > G (p.Met9Arg)	∼0	Pathogenic
Frameshit			
4	c.559_562ACAG(p.Asp188fs)	∼0	Pathogenic
5	c.556_559del (p.Lys186fs)	∼0	Pathogenic
6	c.414_417dup (p.Met140fs)	∼0	Likely pathogenic
7	c.120del (p.Val42fs)	∼0	Pathogenic
Nonsense			
8	c.451C > T (p.Gln151Ter)	∼0	Pathogenic
Splice site			
9	c.343+1G > A	0.000109	Pathogenic

## Discussion

We identified a mainland Chinese family with the recurrent homozygous c.509G > A variant in *PPIB* that causes OI type IX. This variant can now be classified as “Pathogenic.” Including our case, this variant has been detected in four independent families, all of Chinese origin, and the variant is absent from other populations in gnomAD; thus, this is likely a Chinese-specific pathogenic variant.

Ethnic specific variants had been reported for other autosomal recessive OI genes. For example, c.321_353del in *FKBP10* was only detected in Turkish OI individuals in homozygous status as a founder mutation (Alanay et al., 2010). Essawi et al. found a recurrent exon 4 deletion p. (Gly152Alafs*5) in *TMEM38B* with a shared haplotype in 12 probands indicating a founder alteration (Essawi et al., 2018). Besides, *LEPRE1* c.1080+1G > T mutation was also identified as a founder mutation carried by 1.5% of the West Africans and 0.4% of the African Americans (Cabral et al., 2012). The identification of ethnic specific founder mutation can help to determine screening strategy for disease prevention.

By estimating the age of the mutation, we demonstrate that the mutation originated from a founder who could be traced to Chinese ancestors over 60,000 years ago. The mutation was very old. With high AF of two flanking SNVs (chr15:g.64448365 and 64792532), this rare variant is predicted to have occurred on a common haplotype block. Since the average cover of WES for other candidates is smaller than that of the proband. Some positions on the proband’s allele were not detected for the cases from Taiwan and 11 carriers. The shared haplotypes between each of the candidates and proband maybe longer. The estimation of the age of the mutation may be younger, but the minimum shared haplotype is certain (Chr15: 64448365–64792532). This mutation will still be very old and much older than that founder mutation reported in the Chinese population. The Fabry disease-causing mutation, the GLA IVS4+919G > A, was estimated to be originated from a founder mutation that occurred in a Chinese chromosome more than 800 years ago (Liang et al., 2020).

OI is a highly heterogeneous collagen-related disorder whose phenotype ranges from barely detectable to perinatal lethal with dominant. OI-IX is one of the recessive forms due to mutations in peptidyl prolyl cis–trans isomerase *PPIB* gene. *PPIB* plays a vital role in 3-prolyl hydroxylation/rate-limiting complex of collagen I post-translational modification and folding (Marini et al., 2010). Cabral WA et al. found collagen folds more slowly in the absence of CyPB in *PPIB* KO cells and tissues (Cabral et al., 2014).

We estimated the additive PPIB pathogenic variants in EA population based on reported P/LP variant in database to be over 1/1,000. This is certainly a conservative estimation since some missense variants that are eventually found to be pathogenic are not ascertained, and the current data suggest that the *PPIB* gene can be included in expended carrier screening so that this severe condition can be prevented at an early stage.

We concluded that the recurrent c.509G > A/p.G170D variant in *PPIB* is pathogenic, and likely, a Chinese founder mutation can be traced back to over 60,000 years ago. Identification of such founder mutation provides important information to preventive testing and genetic counseling.

## Data Availability

The original contributions presented in the study are included in the article/Supplementary Material, further inquiries can be directed to the corresponding authors.
