# Supplementary material for: A Founder Pathogenic Variant of PPIB Unique to Chinese Population Causes Osteogenesis Imperfecta IX
Source: Front Genet. 2021 Sep 29;12:717294. doi: 10.3389/fgene.2021.717294 (PMC8511635; doi:10.3389/fgene.2021.717294)
Supplement: Supplementary file 1 [file DataSheet1.PDF]

Supplementary Material

1 Supplementary Figures and Tables

Supplementary Figure 1. The Sanger sequencing of the variant c.509G>A.

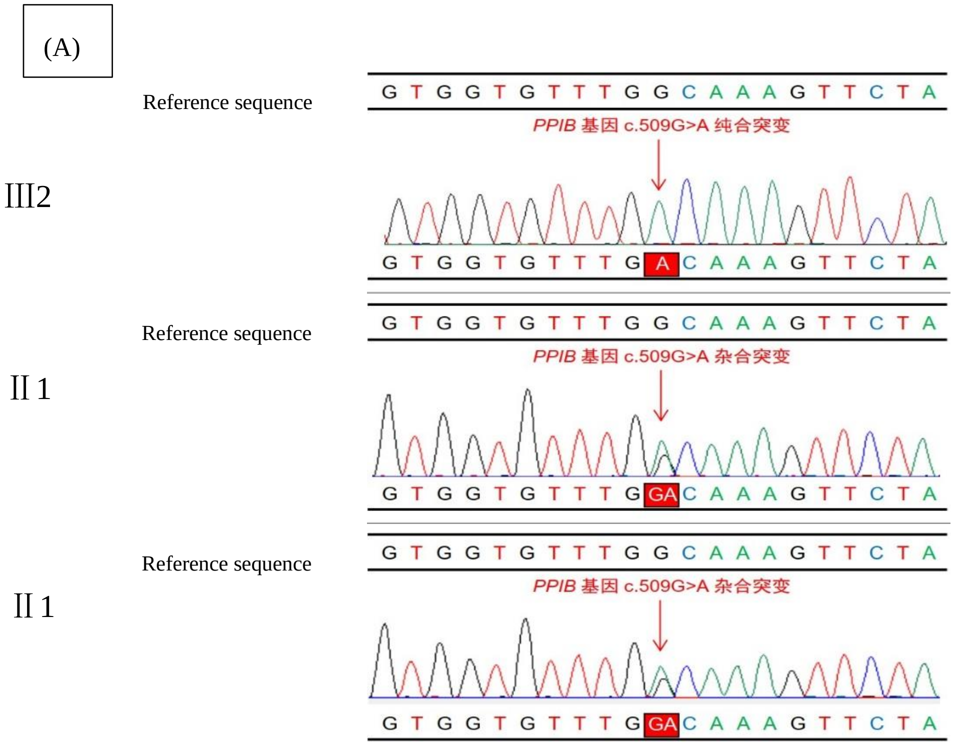

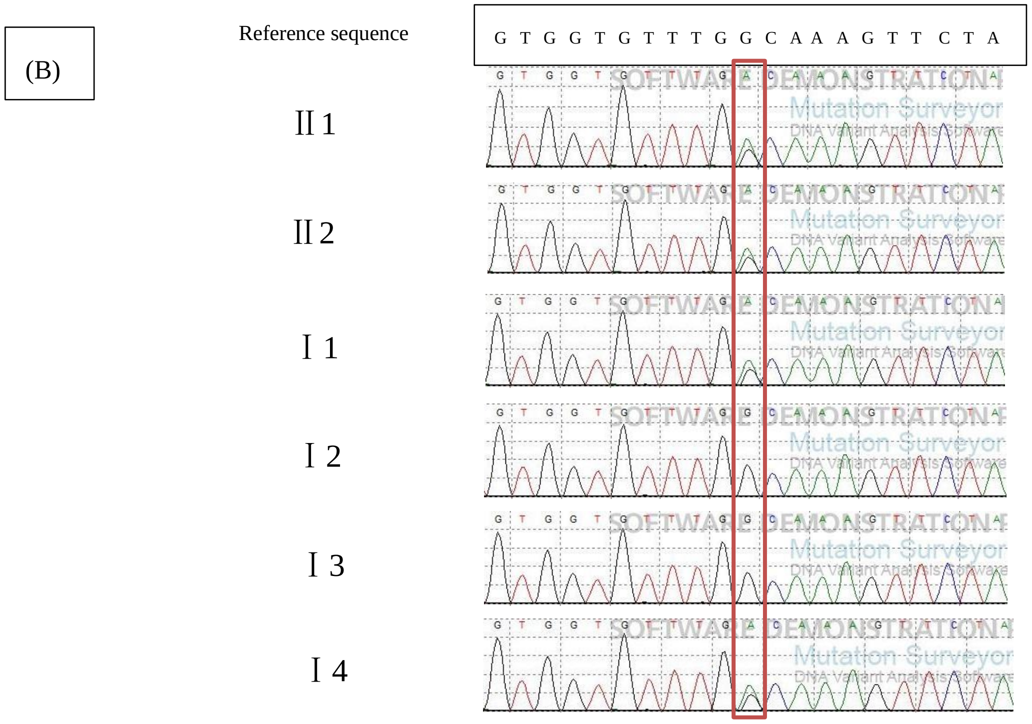

Note: The code for each person is same as that in the figure of pedigree (Fig. 1). The Sanger sequencing of the variant c.509G>A in the proband and its parents (A). The Sanger sequencing of the variant c.509G>A in the parents and grandparents of proband (B).
